# CD44 aptamer mediated cargo delivery to lysosomes of retinal pigment epithelial cells to prevent age-related macular degeneration

**DOI:** 10.1016/j.bbrep.2019.100642

**Published:** 2019-05-01

**Authors:** Chetan Chandola, Marco G. Casteleijn, Urvashi M. Chandola, Lakshmi Narayanan Gopalan, Arto Urtti, Muniasamy Neerathilingam

**Affiliations:** a Department of Lipid Science, CSIR- Central Food Technological Research Institute, Mysore, 570020, India; b Drug Research Program, Division of Pharmaceutical Biosciences, Faculty of Pharmacy, University of Helsinki, Helsinki, 00014, Finland; c School of Pharmacy, Faculty of Health Sciences, University of Eastern Finland, Kuopio, 70211, Finland; d Institute of Chemistry, St. Petersburg State University, Biomedical Chemistry Laboratory, Peterhoff, Universitetskii Pr. 26, 198504, St Petersburg, Russia; e VTT Technical Research Centre of Finland Ltd., Espoo, FI-02044, Finland

**Keywords:** Age-related macular degeneration, CD44 aptamer, Lysosomal delivery, Oxidative stress, RPE cells, Targeted delivery

## Abstract

Age related macular degeneration (AMD) is a progressive, neurodegenerative disorder that leads to the severe loss of central vision in elderlies. The health of retinal pigment epithelial (RPE) cells is critical for the onset of AMD. Chronic oxidative stress along with loss of lysosomal activity is a major cause for RPE cell death during AMD. Hence, development of a molecule for targeted lysosomal delivery of therapeutic protein/drugs in RPE cells is important to prevent RPE cell death during AMD. Using human RPE cell line (ARPE-19 cells) as a study model, we confirmed that hydrogen peroxide (H_2_O_2_) induced oxidative stress results in CD44 cell surface receptor overexpression in RPE cells; hence, an important target for specific delivery to RPE cells during oxidative stress. We also demonstrate that the known nucleic acid CD44 aptamer - conjugated with a fluorescent probe (FITC) - is delivered into the lysosomes of CD44 expressing ARPE-19 cells. Hence, as a proof of concept, we demonstrate that CD44 aptamer may be used for lysosomal delivery of cargo to RPE cells under oxidative stress, similar to AMD condition. Since oxidative stress may induce wet and dry AMD, both, along with proliferative vitreoretinopathy, CD44 aptamer may be applicable as a carrier for targeted lysosomal delivery of therapeutic cargoes in ocular diseases showing oxidative stress in RPE cells.

## 1 Introduction

Age related macular degeneration (AMD) is a progressive, neurodegenerative disorder that leads to the loss of central vision in elderly people [1]. The hallmarks of the disease include age related retinal changes in photoreceptors, retinal pigment epithelium (RPE), and Bruch's membrane. The health of RPE cells, which plays an important role in maintenance and homeostasis of the retina, is critical for the onset of AMD. AMD is classified to two forms: the wet form involves choroidal neovascularization (CNV), and the dry form is associated with geographic atrophy (GA). Either of the two forms may eventually lead to the death of RPE cells, which ultimately results in the death of photoreceptors and gradual loss of vision. Though AMD is a multifactorial disorder, the most common reason for dry AMD is oxidative stress whereas the wet form is mainly caused by inflammation, which may itself be induced by oxidative stress [1]. This oxidative stress is generated in RPE cells due to substantial consumption of oxygen, accumulation of lipid peroxidation products from ingested photoreceptor outer segments, and constant exposure to light. Clinical studies have also shown that dry AMD may develop into wet AMD, hence, it is important to develop therapeutic interventions at the early stage of dry AMD to avoid progression to the more aggressive wet form of the disease.

Lysosomal activity is crucial for the health of RPE cells since it degrades aggregated or misfolded proteins [2]. Reduced lysosomal activity and chronic oxidative stress in the RPE cells was observed in clinical AMD cases [3]. Hence, restoring the lysosomal activity of the RPE cells to minimize AMD pathogenesis was earlier suggested [2] and experimentally proven in the cultured RPE cells by Subrizi et al. [4]. Thus, targeting therapeutic proteins/drugs to lysosomes of RPE cells for overcoming lysosomal inactivity is an important aspect of ocular drug delivery for the treatment of AMD. Even though several attempts have been made earlier for drug delivery into the RPE cells using nanoparticles [5], there is a dearth of systems that specifically target a drug to RPE.

The CD44 receptor is a cell surface glycoprotein which is known to be upregulated in some cancers [6], however its expression in RPE cells has been sparsely studied. The CD44 receptor is not expressed in post-mitotic RPE cells [7], but its upregulation in the RPE during inflammation has been seen in a laser induced CNV rat model [8], and in the RPE cells of fibrotic human retina [7]. The overexpression of cell adhesion molecules, including CD44, in RPE cells has been linked to the initiation of choroidal neovascularization in the laser induced CNV rat model [8]. Interestingly, overexpression of CD44 due to oxidative stress has been observed in a gastric epithelium cell line [9], though, no conclusive study has been conducted to study the direct effect of oxidative stress on CD44 expression in RPE cells *in vitro* or *in vivo*. Since oxidative stress is the underlying cause of dry AMD and one of the factors responsible for multifactorial wet AMD, we decided to study the effect of oxidative stress on CD44 expression in the RPE cells, so that it may be used as a suitable target during oxidative stress for receptor mediated selective drug delivery with a previously reported nucleic acid CD44 aptamer - specific for hyaluronic acid binding domain (HABD) of the CD44 protein [10]. HABD is a conserved domain that is found in all the splice variants of CD44 gene. Aptamers are small, structurally distinct oligonucleotides that exhibit specific and high binding affinities to proteins and other biological macromolecules because of their distinct 3D folding [11]. Due to their ability for easy chemical modification, ease of synthesis, low immunogenicity, low batch-to-batch variation and high stability they are attractive ligands in the field of targeted therapeutics and diagnostics.

The objective of this work was to develop a delivery method that targets the RPE cells and, in particular, lysosomes within the RPE to overcome lysosomal inefficiency. We investigated this CD44 receptor-based system as a potential cell surface target molecule for targeting drug(s) to the diseased RPE cells, specifically during the oxidative stress. The cellular uptake of this system is based on specific receptor-mediated endocytosis of the ligand-label or ligand-drug conjugate. We demonstrate here the proof-of-concept for CD44 based targeted delivery into the diseased RPE cells.

## 2 Material & methods

### 2.1 Cell culture

ARPE-19 cells (human retinal pigment epithelial cell line, ATCC CRL-2302) were cultured as explained earlier [4]. For experiments where differentiated ARPE-19 cells were required, cells were seeded at high density (1.5 × 10^5^ cells/cm^2^) in a low serum media (2% FBS). Cells were allowed to differentiate for 4 weeks at 37 °C in 7% CO_2_ in a humidified incubator. Other constituents were kept the same. MDA-MB-231 and NIH-3T3 cells were grown in DMEM-F12 and DMEM media, respectively, supplemented with 10% FBS, 2 mM l-glutamine and antibiotics at 37 °C in 5% CO_2_ incubator.

### 2.2 Protein extraction and western blotting

Cells were harvested in lysis buffer (50 mM Tris, pH 7.6, 150 mM NaCl, 0.1% SDS, 1% NP-40, and 2 mM EDTA + protease inhibitor cocktail (Roche)) followed by sonication (5 min, medium power) and 15-min incubation on ice for cell lysis. Next, centrifugation was performed for 15 min at 14000 g and supernatant containing total cell extract (TCE) was collected for study. Protein concentration was measured by Bradford assay (Bio-Rad) with BSA as a standard. TCE was loaded on 10% acrylamide gel followed by SDS-PAGE. Rabbit anti-CD44 (1:1000; Abcam) and mouse anti–β-actin (1:2000; Cell signaling) antibodies were used with HRP-conjugated secondary antibodies for western blotting, and the blots were imaged using the Biorad Image Lab imaging system. Blots were cut to enable blotting for multiple antibodies. Densitometric analysis was performed using ImageJ and statistical analysis was performed using Prism6 software.

### 2.3 CD44 expression during oxidative stress

ARPE-19 cells (1.5 × 10^5^ cells/cm^2^) were seeded in a flat bottom 6 well plate (Nunc) and allowed to differentiate for 28 days in a low serum (2% FBS) media. Hydrogen peroxide (Sigma cat. no. 516813) was added to cells grown in above described media at an increasing concentration of 0, 0.50, 0.75, 1, 1.25, 1.5 and 2 mM, respectively, to induce oxidative stress. After 24 h of introducing oxidative stress, the old media was replaced with fresh dilutions of H_2_O_2_ and kept for 24 h more. Thus, oxidative stress was given for a total of 48 h. Total cell extract was prepared as described above and stored in −20 °C freezer until use. Total cell extract was loaded in equal quantity (30ug) on 10% acrylamide gel for SDS-PAGE. Western blot was performed as described above.

### 2.4 Aptamer surface-binding/internalization in cell lines

Proliferating ARPE-19 cells along with CD44 positive (MDA-MB 231) and CD44 negative (NIH-3T3) cell lines were seeded on glass coverslips placed in 24 well plates (Nunc) a day before experiment. All the three cell lines were treated with either FITC-CD44 aptamers or FITC-scramble aptamers (200 nM final concentration) for 90 min at 37 °C in a CO_2_ incubator to allow binding and/or internalization of aptamers. Subsequently, cells were rinsed once each with ice-cold PBS followed by 0.5 N NaCl (ice-cold) to remove unbound or weakly bound FITC-CD44 aptamer from cell surface. Next, a PBS wash was given followed by fixation using 4% paraformaldehyde for 10 min. For visualization of CD44 glycoprotein cells were permeabilized with 0.1% Triton for 10 min, followed by blocking (5% BSA in 0.1% Triton X-100) for an hour. CD44 primary Ab (Abcam) in blocking solution was incubated with cells at 4 °C overnight followed by washing with PBS. Finally, cells were incubated with Alexa-594 labelled goat anti-mouse secondary antibody (Cell Signaling) for an hour at room temperature. Nucleus was counterstained with Hoechst. Imaging was done using Leica DM6000 microscope using 20× objective with an optical magnification of 1.6X. Cells showing aptamer labeling were counted manually using ImageJ software. Speckles (green) showing CD44-FITC aptamer were counted from each field, and divided with total number of cells in the same field (i.e., denoted by stained nuclei), thus, giving average aptamers/cell count. The Graph plotted represents quantitative analysis of surface bound or internalized scramble- and CD44-aptamers for each cell line from an experiment performed in triplicate. n = ≥ 100 cells from 4 different fields.

### 2.5 FITC-CD44 aptamer intracellular distribution

Proliferating ARPE-19 cells were seeded on glass coverslip placed in 24-well plate (Nunc) at a density of 5.2 × 10^4^ cells/cm^2^ two days before the uptake experiment. 24 h post cell seeding, lysosomes and late-endosomes were stained with CellLight Lysosome/Late Endosomes-RFP reagent (Molecular Probes) following the manufacturer's protocol and the cells were returned to the incubator for 16 h. On the day of experiment, cells were incubated with FITC-CD44 aptamer (200 nM) for 90 min at 37 °C in a 7% CO_2_ incubator. Subsequently, the cells were rinsed once each with ice-cold PBS followed by 0.5 N NaCl (ice-cold) to remove unbound or weakly bound FITC-CD44 aptamer. Again a PBS wash was given followed by fixation using 4% paraformaldehyde for 10 min at room temperature. Cells were again washed with PBS, treated with Hoechst for nuclear staining and mounted using antifade diamond mounting media (Molecular Probes). Imaging was done by Leica TCS SP5 confocal microscope using 60× (glycerol) objective.

## 3 Results

### 3.1 CD44 receptor is overexpressed during oxidative stress in RPE cells

**Fig. 1:**
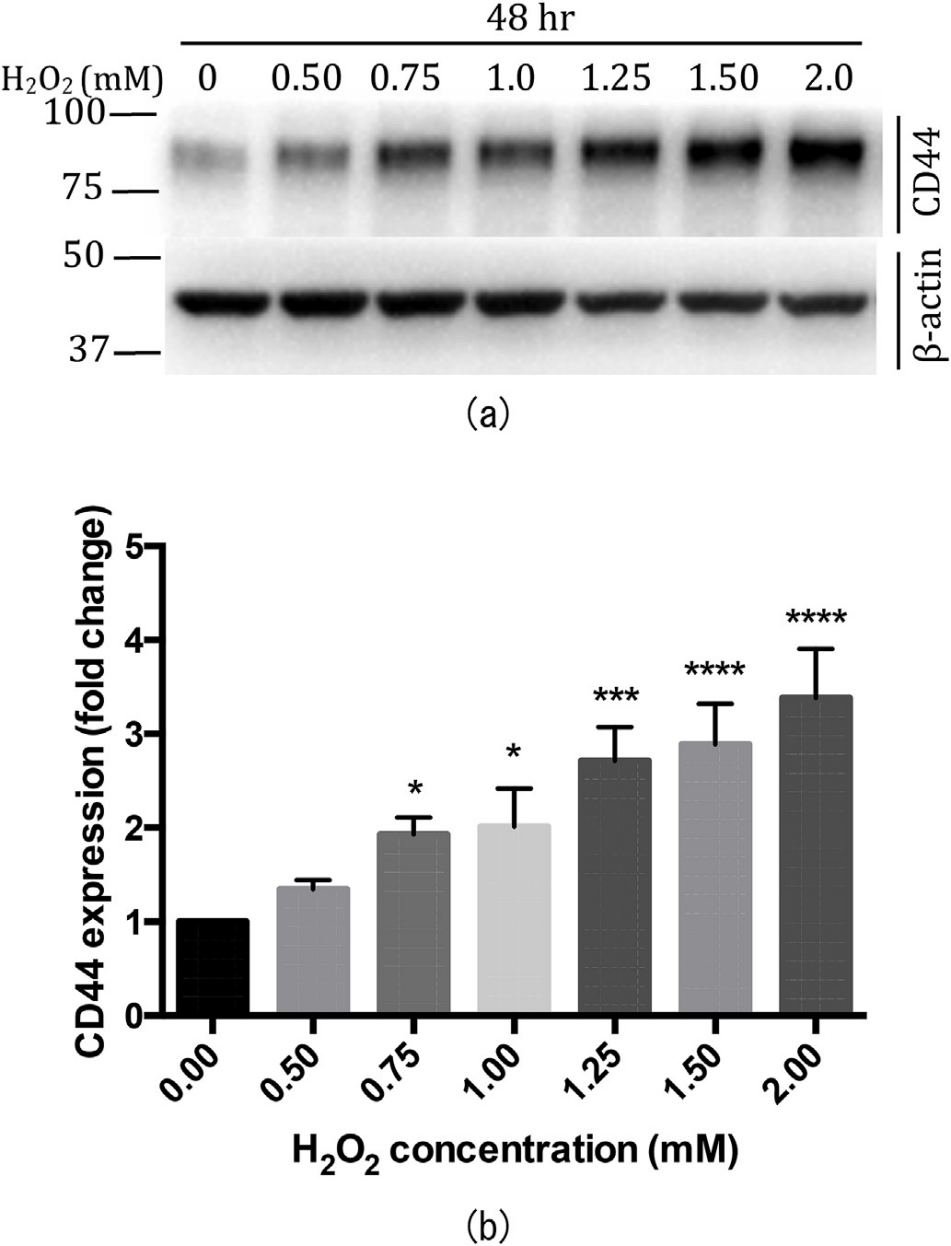
**Upregulated CD44 expression due to oxidative stress in ARPE-19 cells.** Differentiated ARPE-19 cells (DIV28) were treated with increasing concentration of H_2_O_2_ (0, 0.50, 0.75, 1.0, 1.25, 1.50, and 2.0 mM) for 48 h (a) Figure shows cropped blot that is a representation of three independent experiments. Blots from a single membrane were cut after protein transfer, and incubated with different antibodies for evaluation. All gels were run in the same experimental conditions (see material and methods for details) (Full-length blots of each tested protein are reported in Supplementary Fig. S3). WB result shows increasing level of CD44 protein expression with increase in H_2_O_2_ concentration. CD44 expression is determined by anti-CD44 antibody, and β–actin is used as a loading control (b) Graph represents increase in CD44 expression in H_2_O_2_ treated ARPE-19 cells in comparison to untreated cells (0 mM). Untreated (0 mM) cells were used to normalize treated cells (0.50, 0.75, 1.0, 1.25, 1.50 and 2.0 mM) to obtain the fold change in CD44 expression. Statistical analysis is performed using Prism6 software. Histogram is the mean ± standard deviation of three independent experiments. p-value displayed was calculated using ordinary one way ANOVA followed by Dunnett's multiple comparisons test, with a single pooled variance. * = p ≤ 0.05 is considered statistically significant, n = 3. DIV – Days *in vitro*, WB - Western blot.

CD44 surface receptor is a well-known biomarker for certain cancers and cancer stem cells, however its expression pattern has not been studied in the RPE cells under oxidative stress, which is an important factor in the development of AMD. Hence, we validated CD44 cell surface receptor as a suitable target molecule for targeted delivery to RPE cells under oxidative stress. For this purpose a human RPE cell line, i.e., ARPE-19 cells were used as a study model. Proliferating ARPE-19 cells are known to express CD44 protein, unlike post mitotic RPE cells which do not express this protein [8,12,13]. This was confirmed by Western blot (WB) analysis (Supplementary Fig. S1). CD44 expressing MDA-MB-231 cells were used as a positive control, and NIH-3T3 cells were used as a negative control. To obtain a more relevant cellular condition we differentiated ARPE-19 cells for 4 weeks in low serum (2% FBS), to downregulate the expression of CD44 receptors (Supplementary Fig. S2). Similar downregulation of CD44 in differentiated ARPE-19 cells has been reported earlier [12]. Next, to induce oxidative stress on differentiated ARPE-19 cells, an increasing concentration of hydrogen peroxide (H_2_O_2_) was added to the cells (0–2 mM for 48 h) followed by Western blot from the total cell extract. H_2_O_2_ concentration at 2.5 mM and above resulted in visible stress and death of ARPE-19 cells - presumably due to oxidative stress, hence, not considered for analysis (Supplementary Fig. S3). Increased oxidative stress, i.e., 0–2 mM H_2_O_2_ resulted in upregulation of CD44 in differentiated ARPE-19 cells (Fig. 1). This observation may be translated to the *in vivo* condition where oxidative stress in ageing RPE cells might lead to an overexpression of CD44 cell surface receptor, in AMD patients.

### 3.2 Specific binding of CD44 aptamer to ARPE-19 cells

To study the specificity of CD44 aptamer to proliferating ARPE-19 cells we compared it with CD44 positive (MDA-MB-231) and CD44 negative (NIH-3T3) cell lines by immunofluorescence. Proliferating ARPE-19 cells – due to constitutive expression of CD44 glycoprotein - were used as an alternative for post-mitotic RPE cells under oxidative stress, as a proof-of-concept model, to confirm the FITC conjugated CD44 aptamer surface binding and/or internalization.

**Fig. 2:**
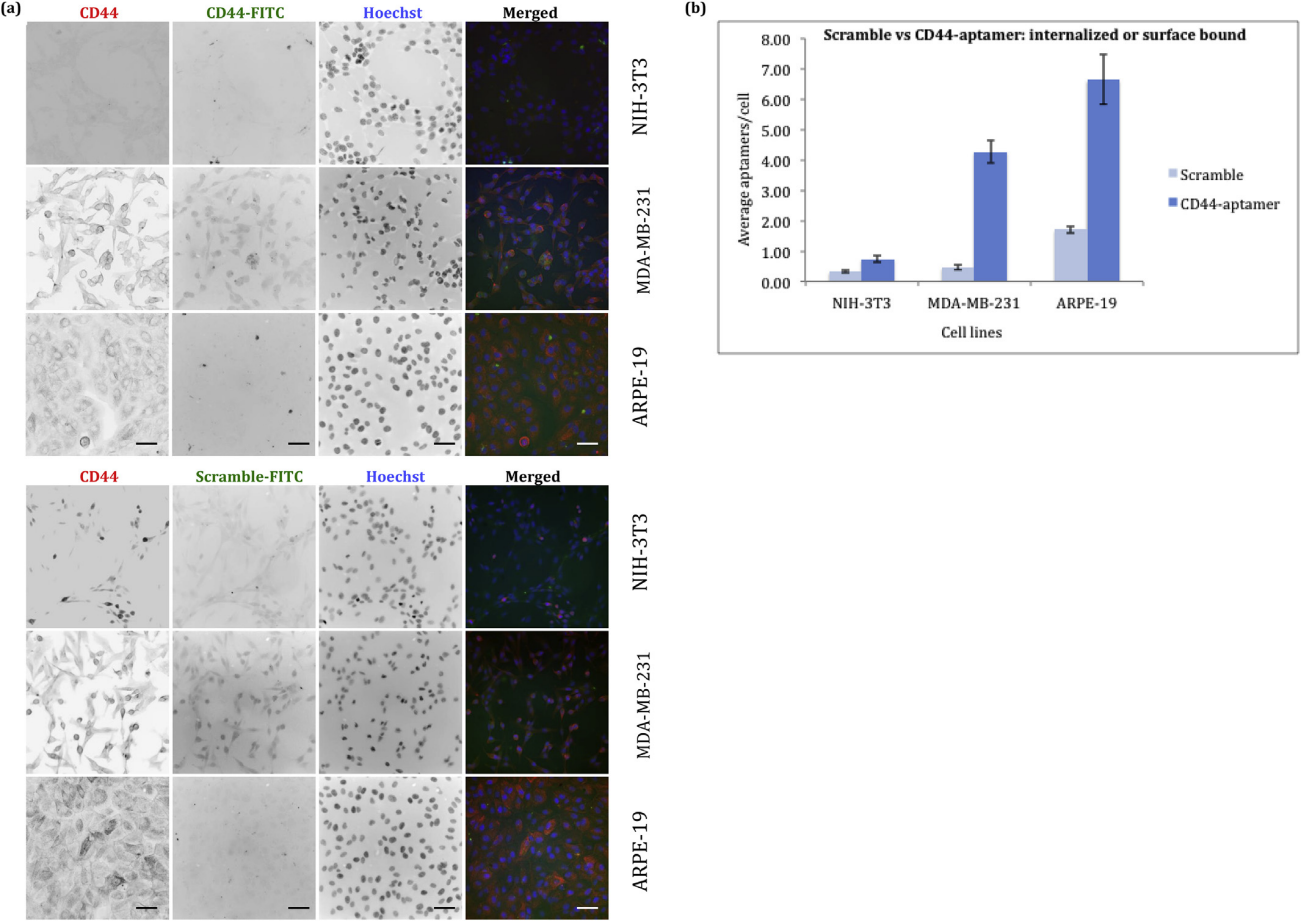
**ARPE-19 cells internalize FITC labelled CD44 aptamer.** ARPE-19 cells, along with CD44 positive (MDA-MB 231) and CD44 negative (NIH-3T3) cell lines, were treated with FITC labelled CD44 aptamers for 90 min to allow surface binding and/or internalization of aptamers. Cells showing aptamer labeling were counted manually. Figure (a) shows NIH-3T3, MDA-MB-231 and ARPE-19 cells that bind or internalize CD44 aptamer (green). CD44 expression in cell lines is shown by labeling with CD44 Ab (red) and nucleus is stained with Hoechst (blue). (b) Graph represents quantitative analysis of internalized or surface bound CD44^−^or scramble-aptamers for each cell line from 4 different fields. n = ≥ 100 cells. Experiment was performed in triplicate. Scale bars are 50 μm.

Here, the fluorescent probe FITC was conjugated as cargo to the aptamer to demonstrate and visualize the cellular delivery of aptamer. Each aptamer is conjugated to single FITC molecule at 5’ terminal. For quantitative analysis, widefield fluorescence imaging was performed. The fluorescent signal (i.e., each signal representing an aptamer) in each cell in a visual field was counted (Fig. 2a). Total number of signal counts were averaged as per cell count from atleast hundred cells (Fig. 2b). Maximum internalization or surface binding of FITC-CD44 aptamer was observed in ARPE-19 cells, presumably due to high CD44 cell surface receptor expression (as shown in Supplementary Fig. S1). Though MDA-MB-231 cells express CD44 receptor, it had less signal as compared to ARPE-19 cells. NIH-3T3 cells showed the lowest signal for CD44 aptamer. Infact, many NIH-3T3 cells had no fluorescent aptamer signal. The signal in some negative control NIH-3T3 cells is probably due to the internalization by non-receptor mediated endocytosis. ARPE-19 cells demonstrated approximately nine-fold internalization of FITC-CD44 aptamers in comparison to negative control NIH-3T3 cells. Scrambled aptamer internalization by NIH-3T3, MDA-MB-231 and ARPE-19 cells was significantly low. Higher internalization of scrambled aptamer by ARPE-19 cells may be explained by the phagocytic nature of the RPE cells as compared to the other cells in this study. However, in ARPE-19 cells CD44-aptamer internalization was four fold higher as compared to scramble aptamer, thus demonstrating the role of CD44 receptor mediated internalization. Hence, this result shows that CD44 aptamer has a potential to deliver conjugated cargo to CD44 positive ARPE-19 cells.

### 3.3 Internalization of CD44 aptamer to lysosomes

**Fig. 3:**
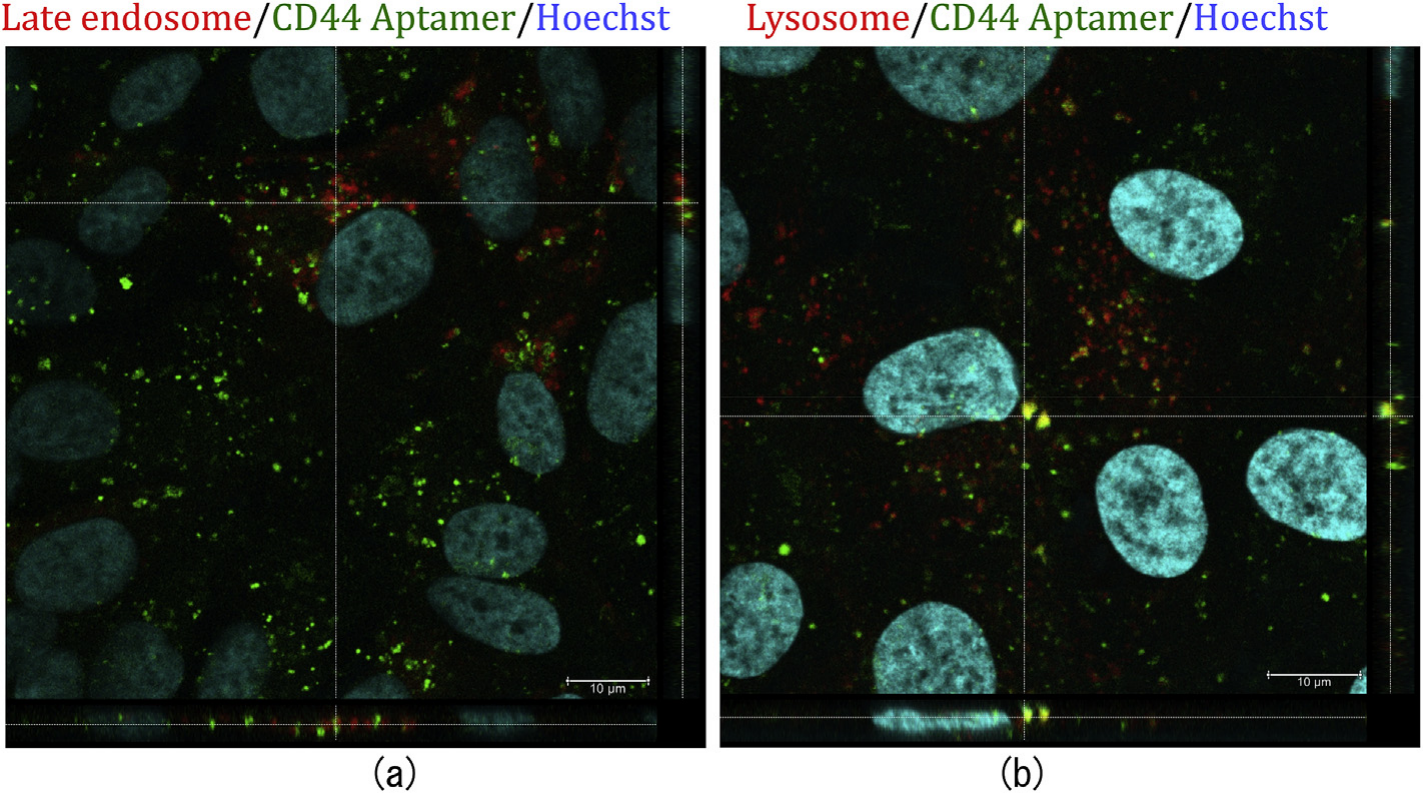
**Intracellular localization of FITC-CD44 aptamer in proliferating ARPE-19 cells.** FITC-CD44 aptamer (green) was incubated with the cells for 90 min, immediately thereafter the cells were rinsed with 0.5 N NaCl followed by fixation. Top view alongwith vertical and horizontal orthogonal sections show colocalization of FITC-CD44 aptamer with late endosomes (a) and lysosomes (b). Late endosomes and lysosomes (red) were stained with CellLight RFP reagent. Nucleus counterstained with Hoechst (blue). Imaging by Leica TCS SP5 confocal microscope using 63× objective. Scale bars are 10 μm.

The intracellular localization of the internalized aptamer is important because it may determine the therapeutic effects of the conjugated cargo. Hence, we studied qualitatively the localization of internalized FITC conjugated CD44 aptamer in proliferating ARPE-19 cells. To this aim, we stained the late-endosomes and lysosomes of proliferating ARPE-19 cells with CellLight Endosome-RFP and CellLight Lysosome-RFP, respectively. Subsequent incubation with FITC-CD44 aptamer showed significant localization of CD44 aptamer (green) in late endosomes and lysosomes (red) (Fig. 3).

## 4 Discussion

Protein misfolding, aggregation and abnormal degradation are central to many neurodegenerative diseases, including AMD. Effective lysosomal activity plays an important role in abnormal protein degradation and, hence, in maintaining the health of RPE cells. However, during chronic oxidative stress RPE cells are known to lose their lysosomal efficiency [3,4]. There is also a dearth of targeting systems that deliver a therapeutic cargo to RPE cells, particularly to lysosomes to overcome the lysosomal inefficiency. The only efforts for targeted delivery of a cargo to RPE cells have been done using hyaluronan [14], however, using hyaluronan for targeted delivery has its own limitations [15]. Here, we show CD44 cell surface receptor as a novel target molecule for RPE cells under oxidative stress, and validate the use of CD44 aptamer for lysosomal targeting in RPE cells to prevent AMD.

This study identifies CD44 cell surface glycoprotein as a novel target molecule for RPE cells that are in oxidative stress since this receptor is not expressed in either fetal or adult post-mitotic human RPE cells. An earlier study shows a contradictory result and demonstrates CD44 transcript downregulation in an acute hyperoxia-induced mouse model by microarray analysis [16]. However, that study used RPE/choroid instead of RPE cells alone derived from a rodent model, and the study was done at transcriptome level which might explain the difference from our results. The same research group earlier studied the effect of oxidative stress on ARPE-19 cells using three different oxidative stress inducing agents, and did not report CD44 among the list of differentially regulated genes [17]. In comparison, we studied the effect of oxidative stress on a differentiated human RPE cell line alone, i.e., ARPE-19 cells at protein level. Earlier studies have discussed the disparity in mRNA and protein level expression in various diseases [18], but the protein levels are the most important in the disease [19]. Overall, it seems that CD44 receptor expression is upregulated in the presence of oxidative stress. For clinical significance, this finding should be confirmed in the eyes of the AMD patients. Since CD44 is absent in post-mitotic human RPE cells, but overexpressed in the pathologic condition (during oxidative stress), it may be used as a target for receptor-mediated delivery of therapeutic agents to RPE cells.

Secondly, this is the first study that shows the ability of CD44 aptamer in targeted delivery of cargo to the lysosomes of RPE cell. Previous studies on targeting molecules for lysosomal drug delivery are either not specific to RPE cells [20,21] or, if specific to RPE cells, the lysosomal delivery was not studied [14,22]. We attached a FITC-fluorophore, analogous to a small molecule drug (e.g. shikonin that elevates chaperone protein expression in cells) [23] to CD44 aptamer, thus, demonstrating the ability of the CD44 aptamer to deliver conjugated molecules to the RPE cells. However, conjugation chemistry that enables the release of the drug from CD44 aptamer in the lysosome should be used for effective lysosomal release [24]. The lysosomal localization of CD44 aptamers is supported by an earlier study as well, where LRP-1 protein facilitated lysosomal localization of CD44 receptor in tumor cells [25]. Possibly, after the binding of CD44 aptamer to CD44 receptor, it might be localized to lysosomes of RPE cells through the LRP-1 dependent mechanism. An earlier work has demonstrated the importance of delivery of a therapeutic chaperone protein to the lumen of lysosomes in overcoming a lysosomal inefficiency disorder [26]. Since RPE cells in AMD also suffer from lysosomal inefficiency, we need an efficient molecule that can deliver the drugs to lysosome lumen efficiently and we demonstrate the applicability of CD44 aptamer for this purpose. Hence, localization of CD44 aptamer to lysosomes is of high significance since it may help in targeted delivery of conjugated therapeutic proteins/drugs to the lysosomes of RPE cells during chronic oxidative stress, thus, overcoming lysosomal inefficiency induced pathogenesis during AMD [3]. Also, due to smaller size, it may have better penetration across the inner limiting membrane (ILM).

Hyaluronic acid, a well known ligand of the CD44 receptor is internalized and transported to the lysosomes for degradation [27]. However, the fate of a receptor bound ligand might vary depending on the ligand and its molecular weight. Though we know that CD44 aptamer is localized to the lysosome in ARPE-19 cells, its fate towards degradation is to be studied. However, whether the CD44 aptamer is degraded in lysosomes is not a matter of concern since its role is only to work as a carrier for delivering the conjugated therapeutic cargo to the lysosome. Hence, it would be more important to study whether the conjugated therapeutic cargo i.e., a drug or a therapeutic protein would be functional after targeted delivery to the lysosome - facilitated by CD44 aptamer. This can be verified by a follow-up study where therapeutic efficacy of CD44 aptamer-drug conjugate would be studied against a control in RPE cells during oxidative stress *in vitro*. Though liposomes and nanoparticles are known to localize in lysosomes of RPE in some studies, CD44 aptamer may increase their bioavailability and specificity due to receptor-mediated endocytosis. This statement is supported by earlier studies that demonstrate that aptamer conjugation to nanoparticles and liposomes increases their bioavailability in cancer cells [11].

In summary, CD44 aptamer may be an effective carrier molecule for targeted delivery of therapeutic agents to the lysosomes of RPE cells to overcome lysosomal inefficiency during oxidative stress in AMD patients. Also, it might be useful in targeted drug delivery to the RPE cells in other ocular disorders that involve oxidative stress, such as proliferative vitreoretinopathy.

## Author contributions

M.N. conceived the project. C.C., M.N. and A.U. designed experiments. C.C. and U.C. conducted experiments. C.C., M.G.C., LN.G. A.U. and M.N. analyzed data and wrote the manuscript. M.N. and A.U. provided reagents for the project.

## Conflict of interest

The authors declare no conflict of interest.
