# Supplementary material for: CD44 aptamer mediated cargo delivery to lysosomes of retinal pigment epithelial cells to prevent age-related macular degeneration
Source: Biochem Biophys Rep. 2019 May 1;18:100642. doi: 10.1016/j.bbrep.2019.100642 (PMC6500919; doi:10.1016/j.bbrep.2019.100642)
Supplement: Multimedia component 2 [file mmc2.docx]

NIH-3T3

ARPE-19

MDA-MB-231

37

50

75

100


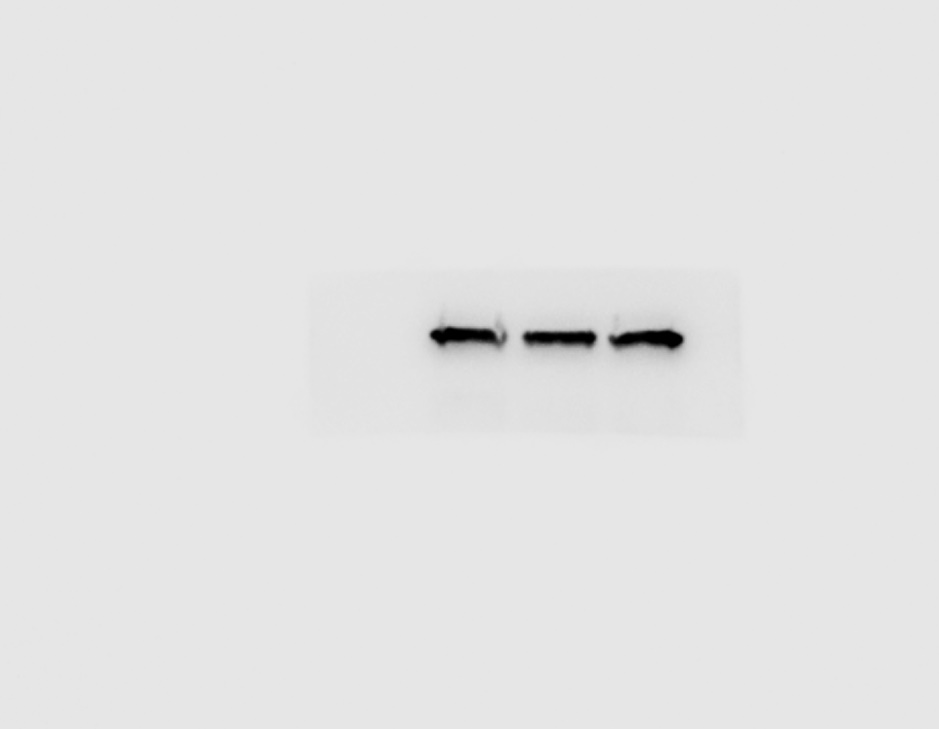

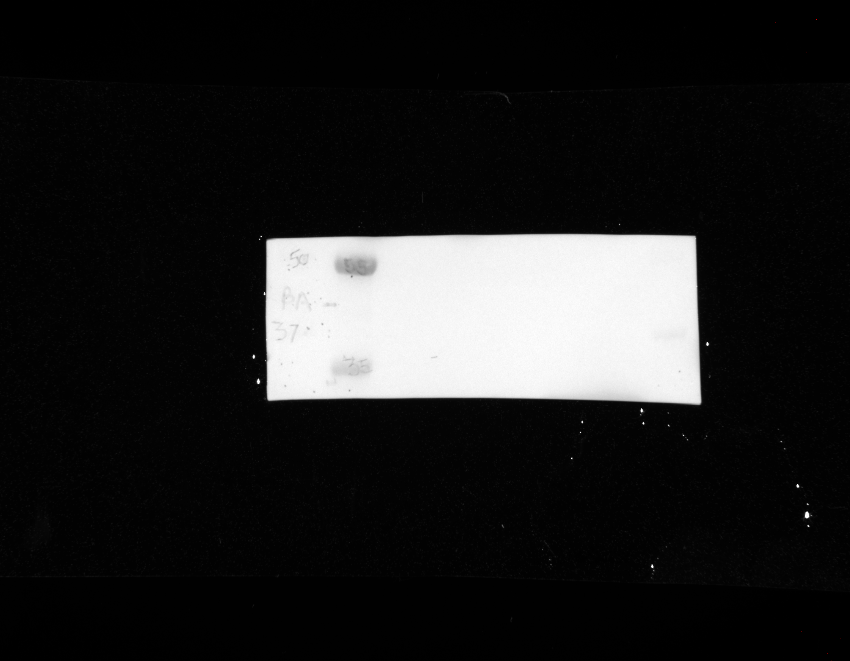

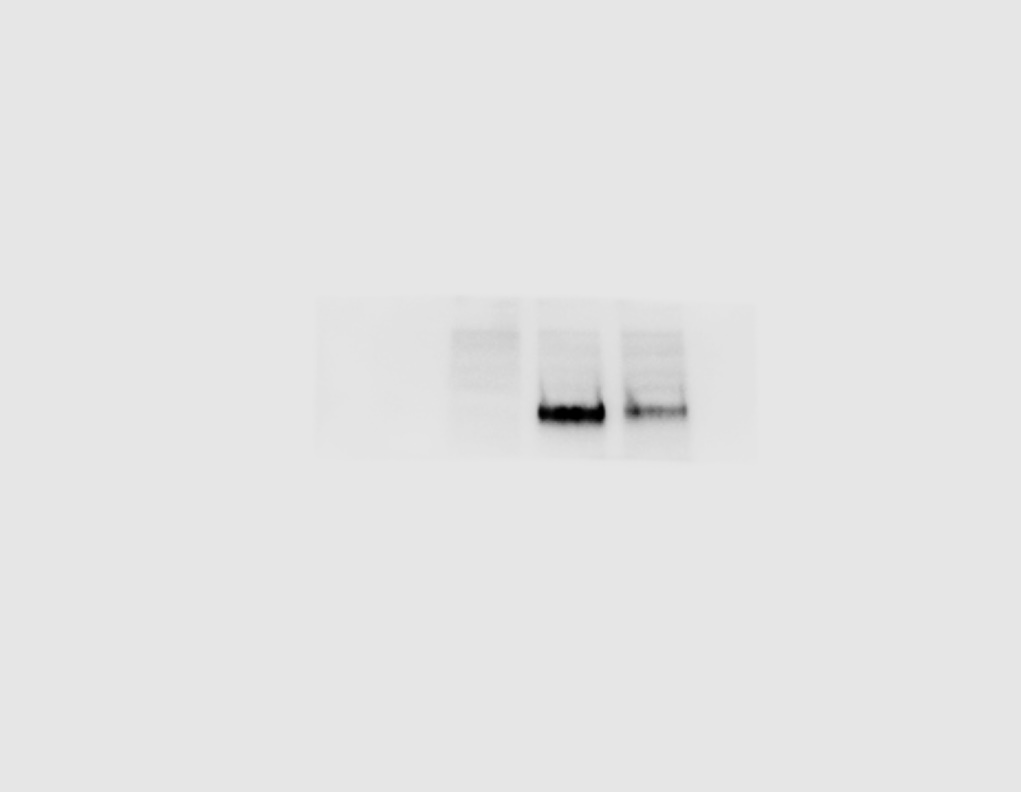

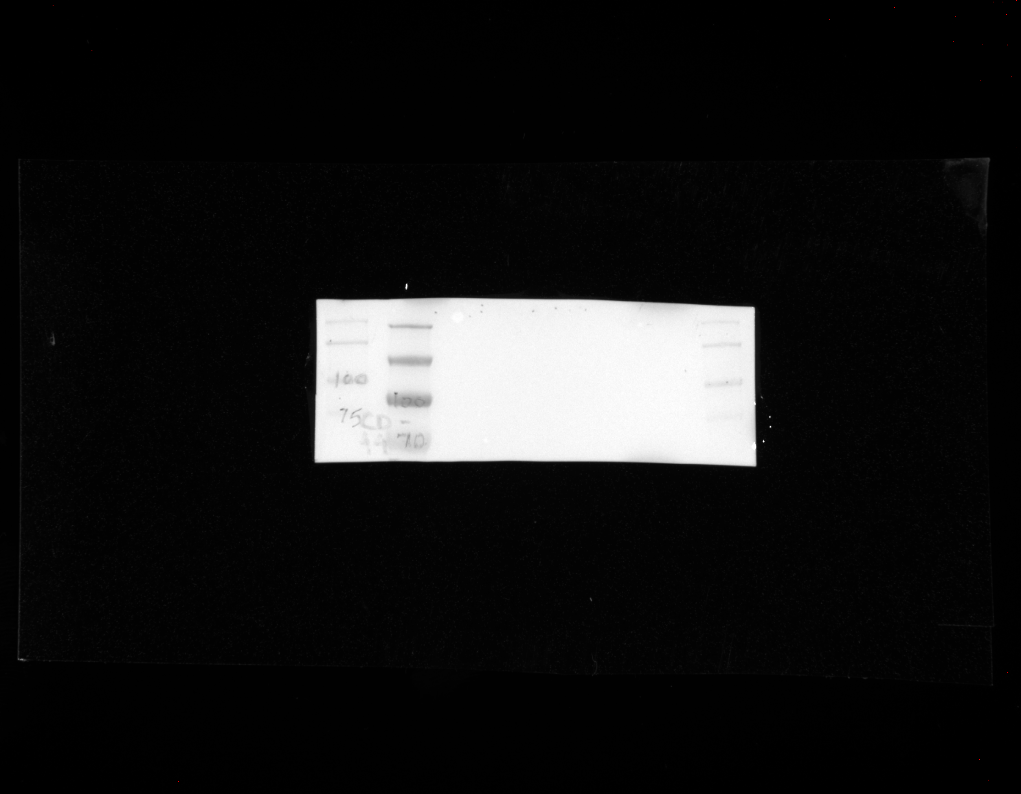


β-actin

CD44

**Supplementary figure S1**


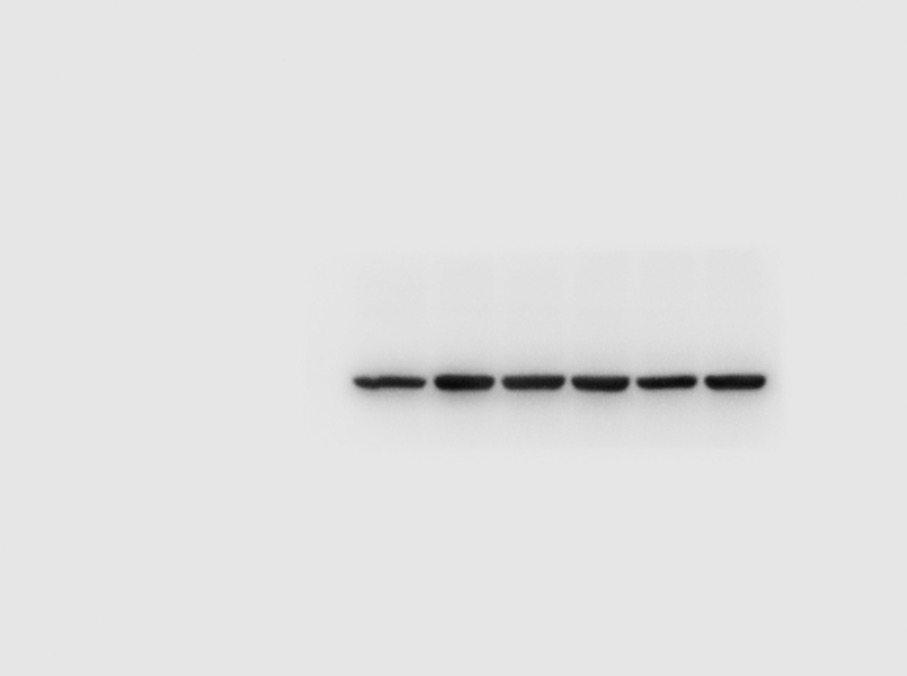

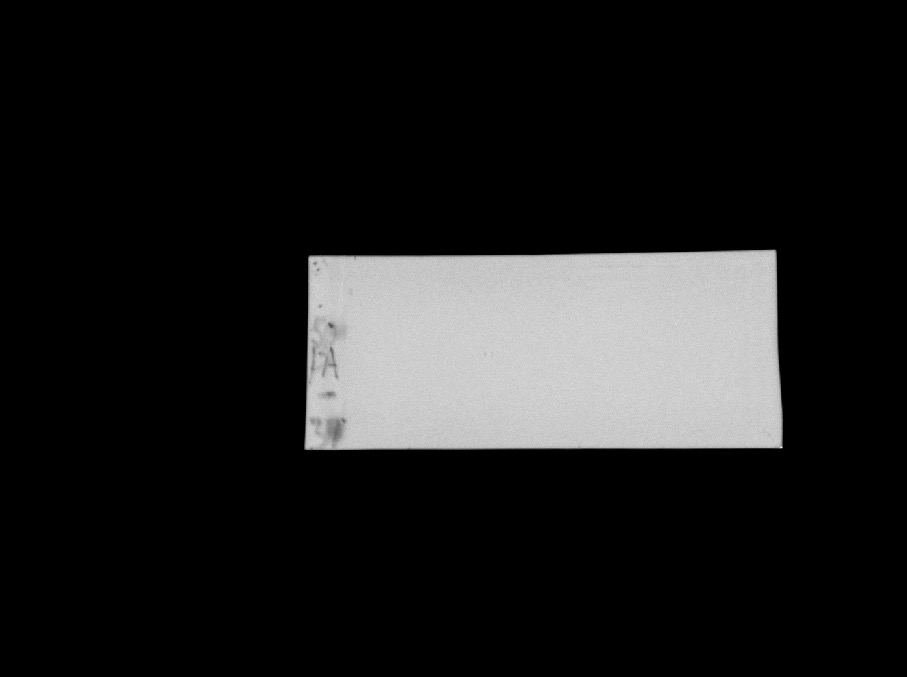

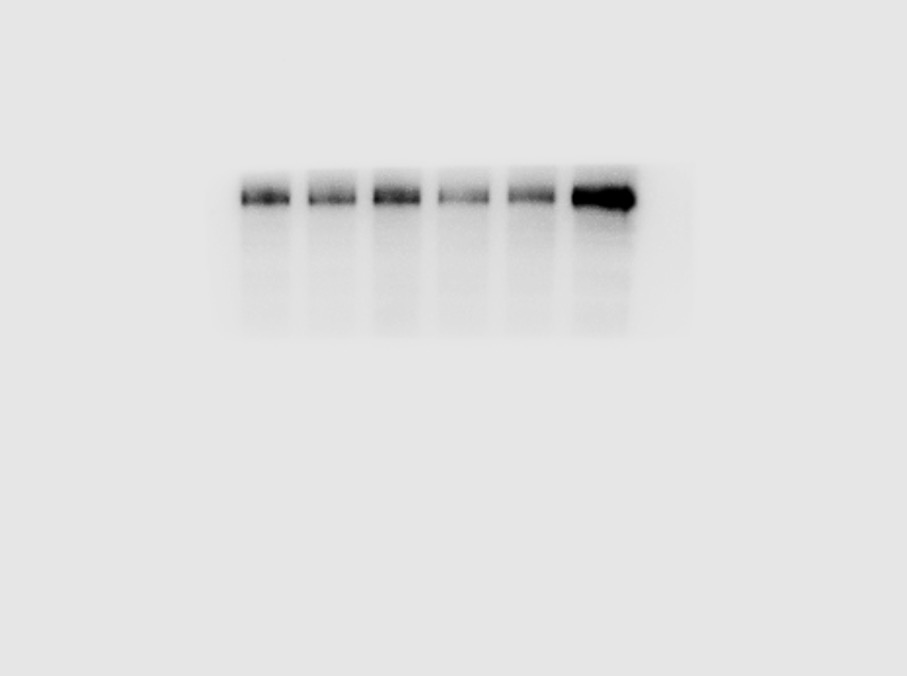

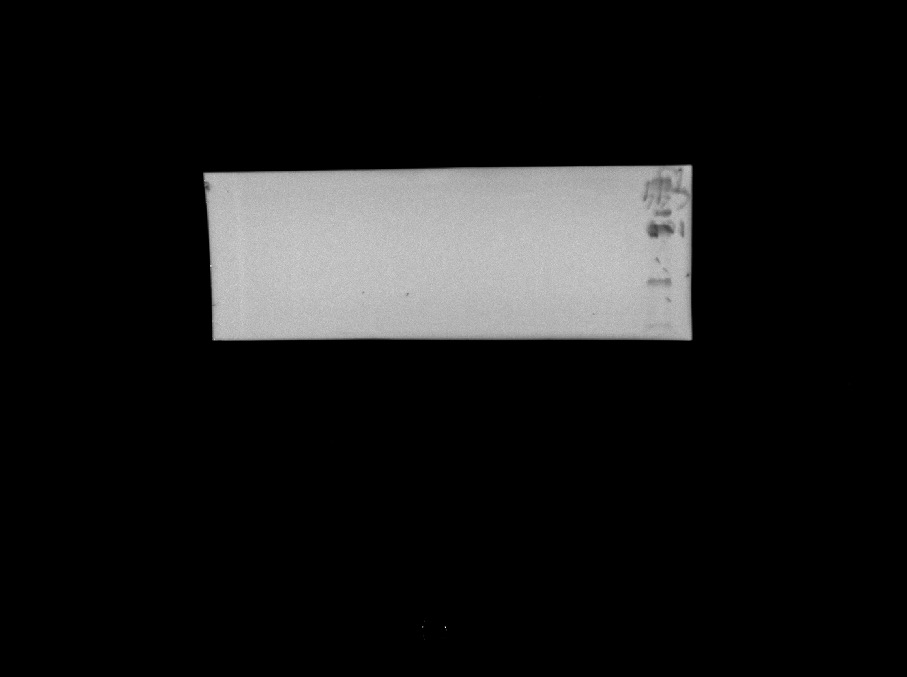


1 7 14 21 28 35

37

50

75

100

DIV

CD44

β-actin

**Supplementary figure S2**


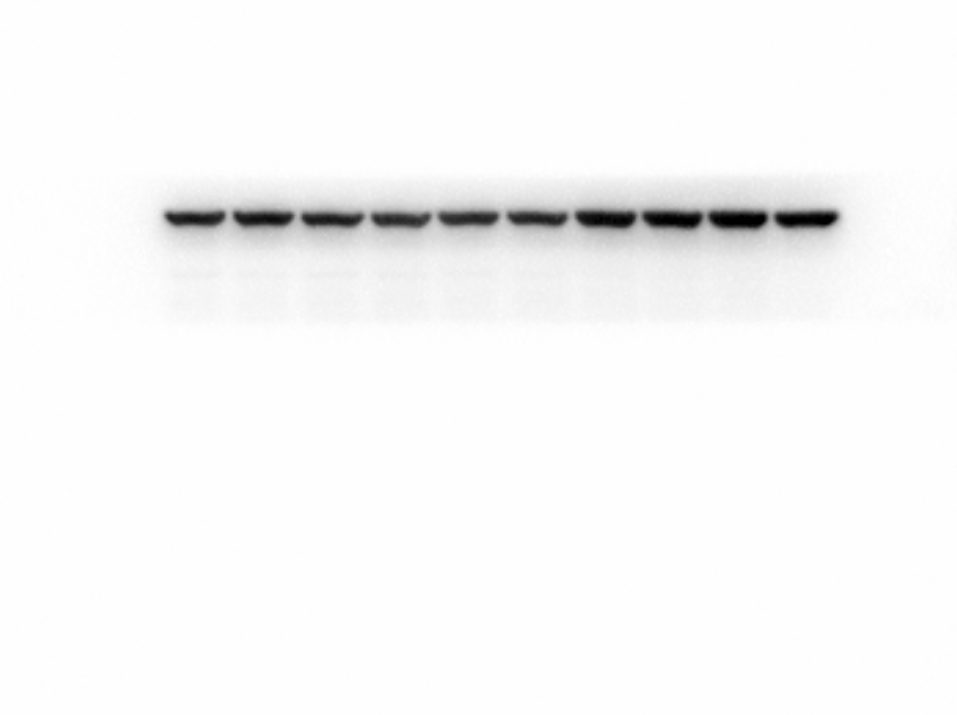

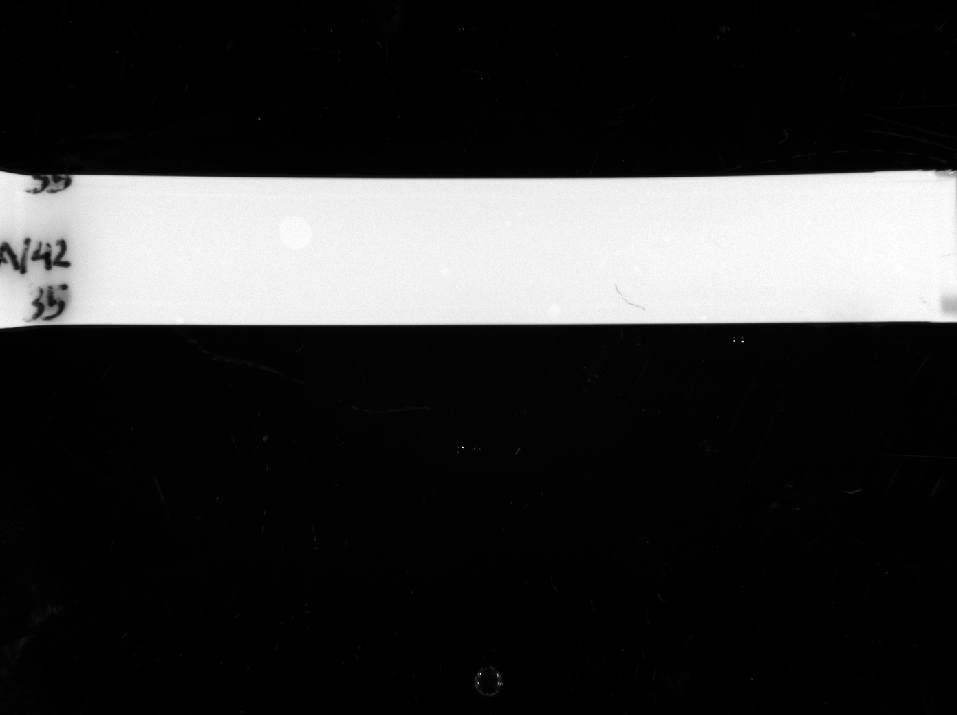

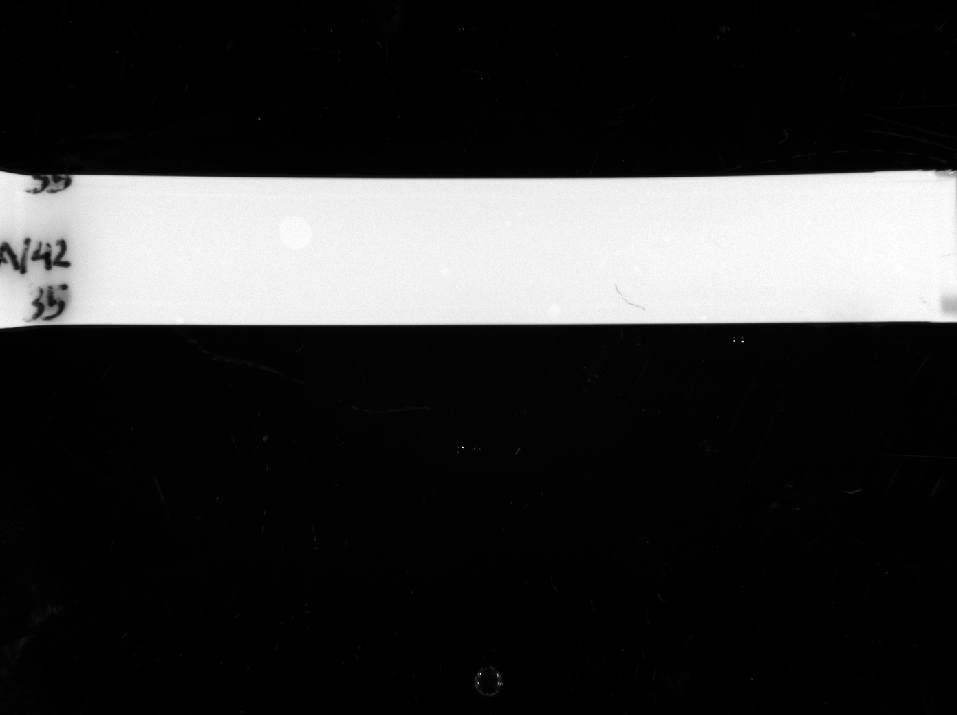

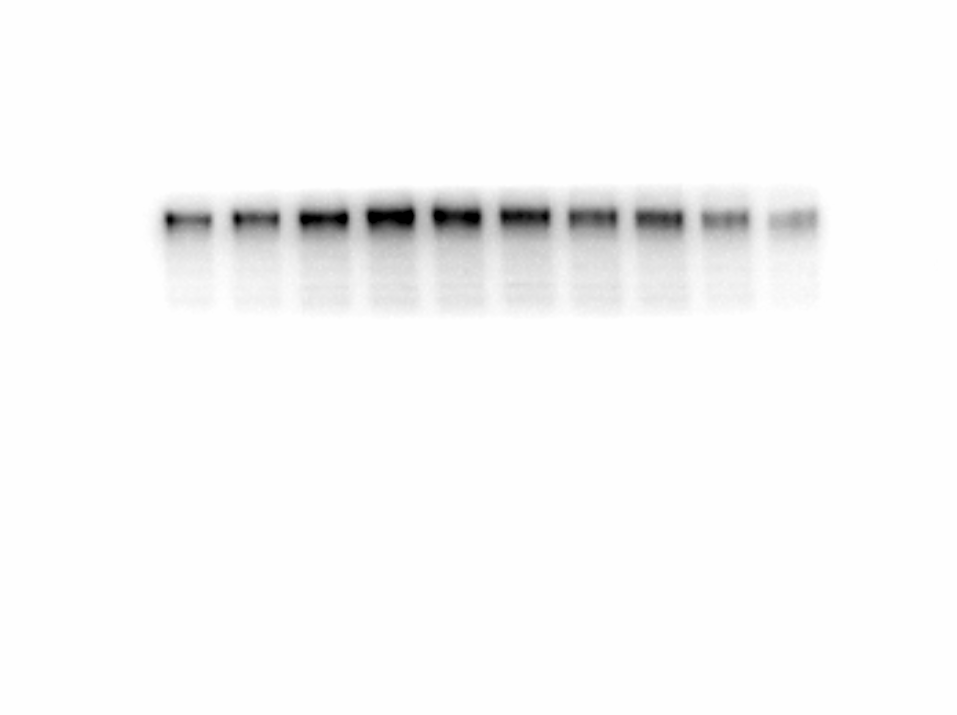

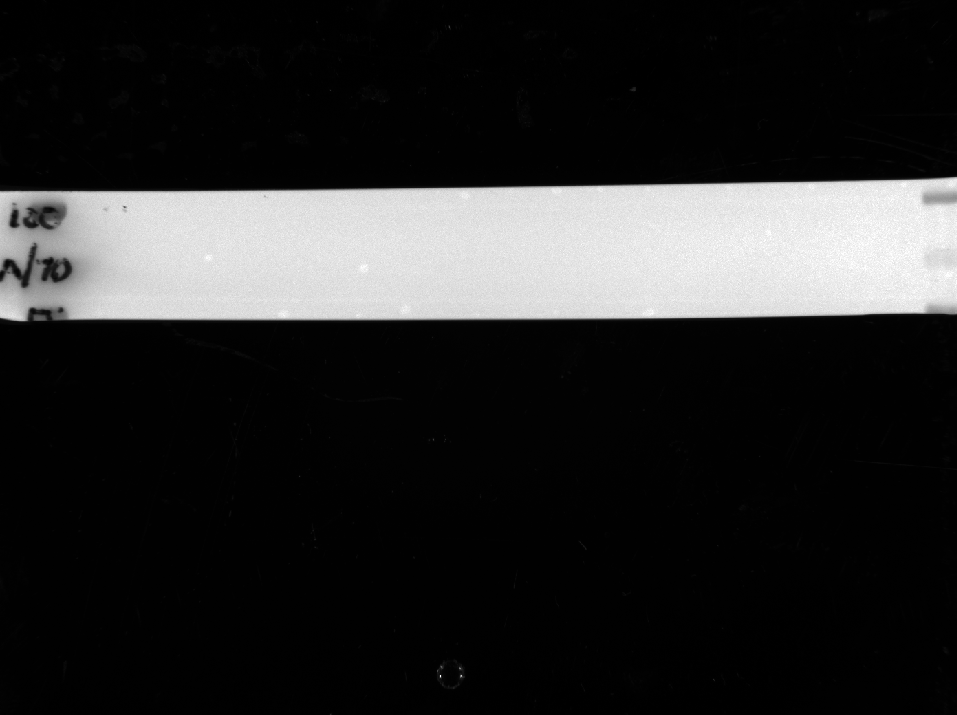

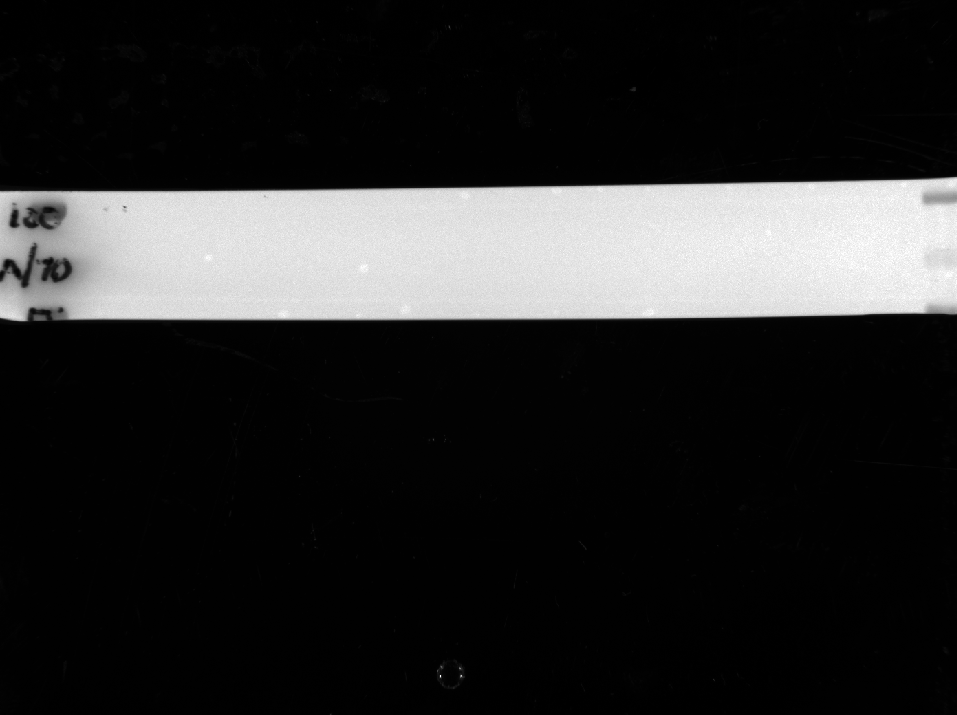


100

75

50

37

CD44

β-actin

0 0.5 0.75 1 1.25 1.5 2 2.5 3 3.5

48 hr

H_2_O_2_ (mM)

**Supplementary figure S3**

**LEGENDS**

**Supplementary figure S1. CD44 expression in cell lines.** Equal amount of TCE (40 ug/well) was loaded for each cell line and immunoblotting was performed using anti-CD44 antibody. β-actin antibody was used to demonstrate equal loading. ARPE-19 cells expressed highest amount of total CD44 protein followed by MDA-MB-231 cells. NIH-3T3 cells did not show CD44 expression. TCE - Total cell extract.

**Supplementary figure S2. Downregulated CD44 level due to differentiation in ARPE-19 cells.** TCE of differentiated ARPE-19 cells at DIV 1, 7, 14, 21, 28 and 35 were loaded on an acrylamide gel for western blot. Anti-CDKL5 Ab shows downregulation of CD44 protein 7 days post differentiation, and remains low thereafter as compared to DIV 1. β-actin demonstrates equal loading of different samples. DIV - Days *in vitro*, TCE - Total cell extract.

**Supplementary figure S3. Corresponding uncropped blots of western blot shown in Figure 1a.** Membrane was cut to enable blotting for multiple antibodies. Western images are overlaid onto image containing MW markers. Red dashed line identifies cropped region. The protein standards are depicted on either side of the blot. H_2_O_2_ at a concentration of 2.5 mM and above lead to visible stress and death of ARPE-19 cells when observed under brightfield microscope just before collecting cells for TCE preparation - presumably due to oxidative stress, hence, not included in analysis. TCE - Total cell extract.

**EQUIPMENTS AND SETTINGS**

For western blot results, the blots were cut and hybridized with different antibodies for immunoblotting. Blots containing prestained molecular weight marker, controls and treated samples were imaged as a single unit using application for colorimetric imaging in Image Lab software (version 5.2.1) supplied with ChemiDoc XRS+ gel doc imaging system (Bio-Rad). Time-lapse chemiluminescence images were captured using the same system. The bright field image of blot was merged with chemiluminiscent image, scaled to same settings, and exported to tiff format file. All the tiff images were cropped using MS Office powerpoint software for figure preparation. Brightness and contrast settings were applied equally across entire image using MS Office powerpoint. Images in supplementary figures display entire blots.

For fluorescent images from Leica DM6000B microscope fitted with Hamamatsu Orca-Flash 4.0 V2 sCMOS camera were used with 20X HC PL APO objective lens with 1.2x optical zoom. Fluorochromes such as Alexa 594, FITC or Hoechst and respective filters were used to capture images using monochromatic camera. Microscope was supported by LAS X software (version 3.4.2). For confocal imaging, we used Leica TCS SP5 confocal microscope with HCX APO 63x (glycerol) lens. Fluorochromes FITC and RFP were excited using Argon (488 nm/35mW) laser while Hoechst was excited using diode (405 nm/50mW) laser. Detection was done by PMTs. Microscope was supported by LAS AF software (version 2.7.7). Images were captured at same exposure settings across all samples of one experiment and preferably in one sitting. All the images were calibrated as per magnification, displayed at same scale settings, color combined using FIJI software and saved as tiff files. The tiff images were cropped and processed equally using FIJI software for figure preparation.
